# Molecular genetic analysis of retinitis pigmentosa in Indonesia using genome-wide homozygosity mapping

**Published:** 2011-11-18

**Authors:** Anna M. Siemiatkowska, Kentar Arimadyo, Luminita M. Moruz, Galuh D.N. Astuti, Marta de Castro-Miro, Marijke N. Zonneveld, Tim M. Strom, Ilse J. de Wijs, Lies H. Hoefsloot, Sultana M.H. Faradz, Frans P.M. Cremers, Anneke I. den Hollander, Rob W.J. Collin

**Affiliations:** 1 Department of Human Genetics, Radboud University Nijmegen Medical Centre, Nijmegen, the Netherlands; 2 Department of Ophthalmology, Radboud University Nijmegen Medical Centre, Nijmegen, the Netherlands; 3 Nijmegen Centre for Molecular Life Sciences, Radboud University Nijmegen Medical Centre, Nijmegen, the Netherlands; 4 Department of Ophthalmology, Diponegoro University, Semarang, Java, Indonesia; 5 Institute of Human Genetics, Helmholtz Zentrum Munchen, Neuherberg, Germany; 6 Division of Human Genetics, Center for Biomedical Research, Faculty of Medicine, Diponegoro University, Java, Indonesia

## Abstract

**Purpose:**

Retinitis pigmentosa (RP) is a clinically and genetically heterogeneous retinal disorder. Despite tremendous knowledge about the genes involved in RP, little is known about the genetic causes of RP in Indonesia. Here, we aim to identify the molecular genetic causes underlying RP in a small cohort of Indonesian patients, using genome-wide homozygosity mapping.

**Methods:**

DNA samples from affected and healthy individuals from 14 Indonesian families segregating autosomal recessive, X-linked, or isolated RP were collected. Homozygosity mapping was conducted using Illumina 6k or Affymetrix 5.0 single nucleotide polymorphism (SNP) arrays. Known autosomal recessive RP (arRP) genes residing in homozygous regions and X-linked RP genes were sequenced for mutations.

**Results:**

In ten out of the 14 families, homozygous regions were identified that contained genes known to be involved in the pathogenesis of RP. Sequence analysis of these genes revealed seven novel homozygous mutations in ATP-binding cassette, sub-family A, member 4 (*ABCA4*), crumbs homolog 1 (*CRB1*), eyes shut homolog (Drosophila) (*EYS*), c-mer proto-oncogene tyrosine kinase (*MERTK*), nuclear receptor subfamily 2, group E, member 3 (*NR2E3*) and phosphodiesterase 6A, cGMP-specific, rod, alpha (*PDE6A*), all segregating in the respective families. No mutations were identified in the X-linked genes retinitis pigmentosa GTPase regulator (*RPGR*) and retinitis pigmentosa 2 (X-linked recessive; *RP2*).

**Conclusions:**

Homozygosity mapping is a powerful tool to identify the genetic defects underlying RP in the Indonesian population. Compared to studies involving patients from other populations, the same genes appear to be implicated in the etiology of recessive RP in Indonesia, although all mutations that were discovered are novel and as such may be unique for this population.

## Introduction

Retinitis pigmentosa (RP), a group of clinically diverse progressive retinal disorders, is a major cause of inherited blindness, affecting 1.5 million people worldwide. RP is initially characterized by night blindness, followed by a gradual loss of peripheral vision and eventually leading to legal blindness [1]. Generally, the manifestation of the first symptom occurs in childhood or early adulthood. The main clinical characteristics of RP are bone-spiculed pigmentations, attenuation of retinal vessels, a waxy pallor appearance of the optic disc, and absent or severely reduced a-waves on electroretinography.

Genetically, RP is also very diverse, with over 50 different causative genes identified to date, and is inherited in an autosomal dominant (30%–40%), an autosomal recessive or isolated (50%–60%), an X-linked (5%–20%), or very rarely in a mitochondrial or digenic manner [2]. Due to the relatively high rate of marriages within specific ethnic groups, autosomal recessive inheritance was reported to be even more frequent in Indonesia [3]. A significant fraction of the causative autosomal recessive RP (arRP) genes have been identified using homozygosity mapping in large, often consanguineous, pedigrees. Recently, we also applied high-resolution homozygosity mapping to identify the genetic defect underlying arRP in non-consanguineous populations [4-7]. Despite a tremendous knowledge on the genetic causes of RP, little is known about the genes involved in RP in the Indonesian population. A study published earlier this year involved the analysis of rhodopsin (*RHO*) in families with an autosomal dominant mode of inheritance [8]. Here we describe the molecular genetic analysis of 14 Indonesian families segregating arRP and show that homozygosity mapping is a powerful tool to identify causative mutations in this population.

## Methods

### Subjects

**Figure 1:**
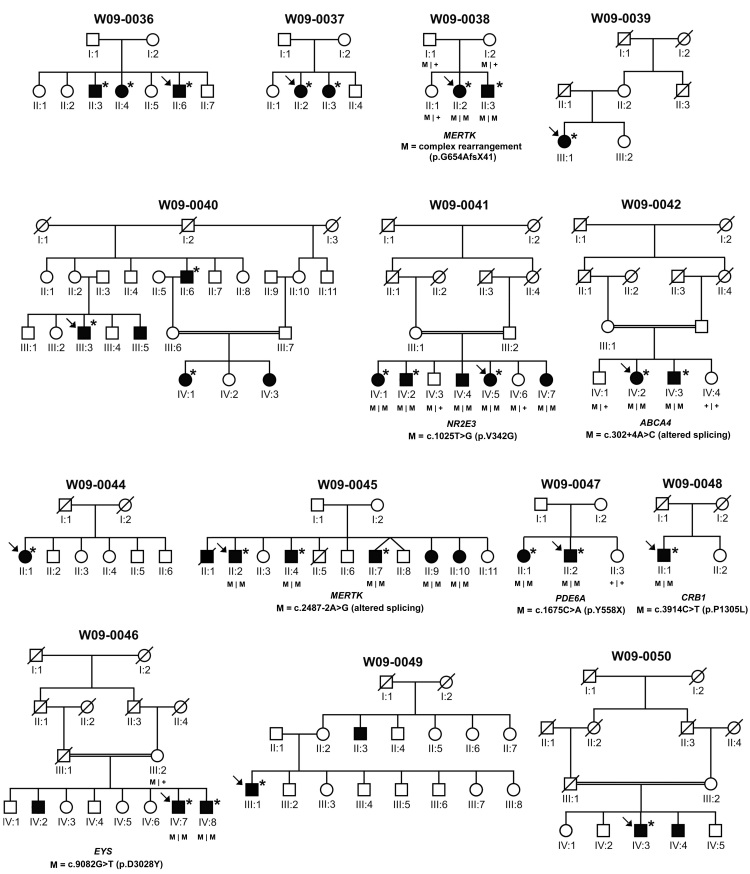
Overview of the pedigree structure of the Indonesian families participating in this study. Affected individuals are indicated with filled symbols, whereas unaffected relatives are indicated by open symbols. Symbols with a slash depict deceased individuals. Probands are indicated with arrows, and individuals that were genotyped on genome-wide SNP arrays are marked with asterisks. Upon the identification of mutations in the probands (gene and mutation indicated below the pedigree), segregation analysis was performed in all available relatives, the results of which are indicated with M (mutated allele) or + (wild-type allele).

Fourteen Indonesian families segregating RP—13 living on Java and one on Sulawesi islands—were enrolled in the study (total number of 56 individuals, 33 affected, 23 unaffected). The affected individuals were 20 males and 13 females of 10–71 years old, with the onset of the disease ranging from the age of 5 to 25. All families appear to be autosomal recessive, except for W09–0049, which is more suggestive of an X-linked recessive pattern. This type of inheritance is also possible in families W09–0048 and W09–0050 (Figure 1). Affected individuals were diagnosed at the Kariadi Hospital, the William Booth Hospital, or the Panti Wilasa Citarum in Semarang Central Java, Indonesia, and examined by direct ophthalmoscopy and visual acuity tests using a Snellen chart. The presumed mode of inheritance was determined by anamnesis and pedigree analysis. Informed consent was obtained from all participating individuals or in the case of under-age participants, from their parents. This study adhered to the tenets of the Declaration of Helsinki. In addition, 149 ethnically matched and unrelated control individuals participated in this study.

### Clinical characterization

Clinical characterization that included funduscopy and visual acuity measurements revealed that the majority of affected individuals had poor vision and showed typical hallmarks of RP on funduscopy, e.g., bone-spicule pigmentation, attenuated retinal vessels, and/or a pale appearance of the optic disc (Table 1). Only in family W09–0036 is the visual acuity not dramatically decreased.

**Table 1:** Clinical and demographical characteristics of affected individuals

**Family code**	**Patient**	**Gender**	**Age of onset (y)**	**Current age (y)**	**Visual acuity**		**Fundus appearance**
					**OD**	**OS**	
W09–0036	II:6	M	21	46	20/40	20/40	AA, BS
	II:4	F	25	50	20/40	20/40	AA, BS
	II:3	M	25	51	20/40	20/40	AA, BS
W09–0037	II:2	F	14	32	HM	HM	AA, BS
	II:3	F	14	29	HM	HM	AA, BS
W09–0038	II:3	M	17	30	HM	CF	AA, BS
	II:2	F	18	32	HM	HM	AA, BS
W09–0039	III:1	F	20	46	LP+	LP+	AA, BS, POD
W09–0040	III:3	M	15	45	LP-	LP+	AA, BS, POD
	IV:1	F	8	10	HM	HM	n.d.
	II:6	M	14	71	HM	LP-	n.d. (cataract)
	III:5	M	13	59	HM	LP-	AA, BS, POD
W09–0041	IV:4	M	14	44	20/200	20/200	BS
	IV:5	F	13	41	20/100	20/70	BS
	IV:1	F	15	49	20/200	20/200	BS
	IV:7	M	15	35	20/40	20/40	BS
	IV:2	M	13	64	HM	HM	AA, BS, POD
W09–0042	IV:2	F	5	64	HM	HM	AA, BS, POD
	IV:3	M	6	62	HM	HM	AA, BS, POD
W09–0044	II:1	F	12	59	LP+	LP+	AA, BS, POD
W09–0045	II:2	M	11	55	LP+	LP+	AA, BS, POD
	II:9	F	11	37	20/240	20/240	BS
	II:10	F	12	35	20/240	20/240	BS
	II:4	M	13	48	HM	HM	BS
	II:7	M	12	39	HM	HM	BS
W09–0046	IV:7	M	14	46	20/400	20/400	BS
	IV:8	M	15	40	20/400	20/400	BS
	IV:5	M	14	47	HM	HM	BS
W09–0047	II:1	F	15	39	HM	HM	BS
	II:2	M	12	33	CF	CF	BS
W09–0048	II:1	M	12	59	LP-	LP-	AA, BS, POD
W09–0049	III:1	M	15	68	LP-	LP-	AA, BS, POD
W09–0050	IV:3	M	15	48	LP+	LP+	AA, BS, POD

Clinical characteristics of all affected family members. M: male; F: female. CF: counting fingers; HM: hand motion; LP+: light perception; LP-; no light perception; AA: attenuated arterioles; BS: bone spicules; POD: pale optic disc; n.d.: not determined.

### Linkage analysis and homozygosity mapping

Genomic DNA of participating individuals was isolated from peripheral blood using a standard salting out procedure [9]. Affected individuals were selected for genome-wide single nucleotide polymorphism (SNP) analysis. For three consanguineous families (W09–0041, W09–0042, and W09–0046), the low-resolution Illumina 6k SNP array (Illumina, San Diego, CA) that contains 6,020 SNPs was used, whereas the patients from the remaining 11 families were genotyped on a high-resolution Affymetrix 5.0 array (Affymetrix, Santa Clara, CA) that contains ~500,000 SNPs. For the three consanguineous families that were analyzed on the Illumina array, multipoint parametric linkage analysis was performed using the GeneHunter 2.1r5 program in the easyLinkage v5.052beta software package [10]. For the other families, homozygous regions were calculated using PLINK software [11], with a cut-off of 3 Mb and allowing two heterozygous SNP calls per window of 50 SNPs.

### Mutation analysis

Exons and intron–exon boundaries of all known arRP genes residing in homozygous regions and X-linked RP genes retinitis pigmentosa GTPase regulator (*RPGR*) and retinitis pigmentosa 2 (X-linked recessive) (*RP2*) were sequenced in the corresponding probands using the Big Dye Terminator Cycle Sequencing Kit v3.1 on a 3130 automated sequencer (Applied Biosystems, Foster City, CA). Upon identification of an unknown variant, available family members and/or ethnically matched control individuals were analyzed using direct sequencing, the amplification refractory mutation system or restriction fragment length polymorphism analysis. Primer sequences and PCR conditions are available upon request. The analysis of mutations was conducted with AlaMut 1.5 software (Interactive Biosoftware, Rouen, France). Splice site scores were calculated using an internal AlaMut module that combines the scores of GeneSplicer, MaxEntScan, SpliceSiteFinder-like, and NNSPLICE. For the missense changes that were identified, the evolutionary conservation of the substituted amino acids was assessed by aligning the corresponding protein sequences of several species, using the Align program in VectorNTI Advance 11.0 software (Invitrogen, Carlsbad, CA). The pathogenicity of these mutations was evaluated using SIFT [12] and PolyPhen-2 [13] prediction programs. In addition, Grantham scores, which define the physicochemical difference between amino acids [14], and PhyloP scores, which are a measure of evolutionary nucleotide conservation (PhyloP44wayAll), were determined.

## Results

### Linkage analysis and homozygosity mapping in arRP families

**Figure 2:**
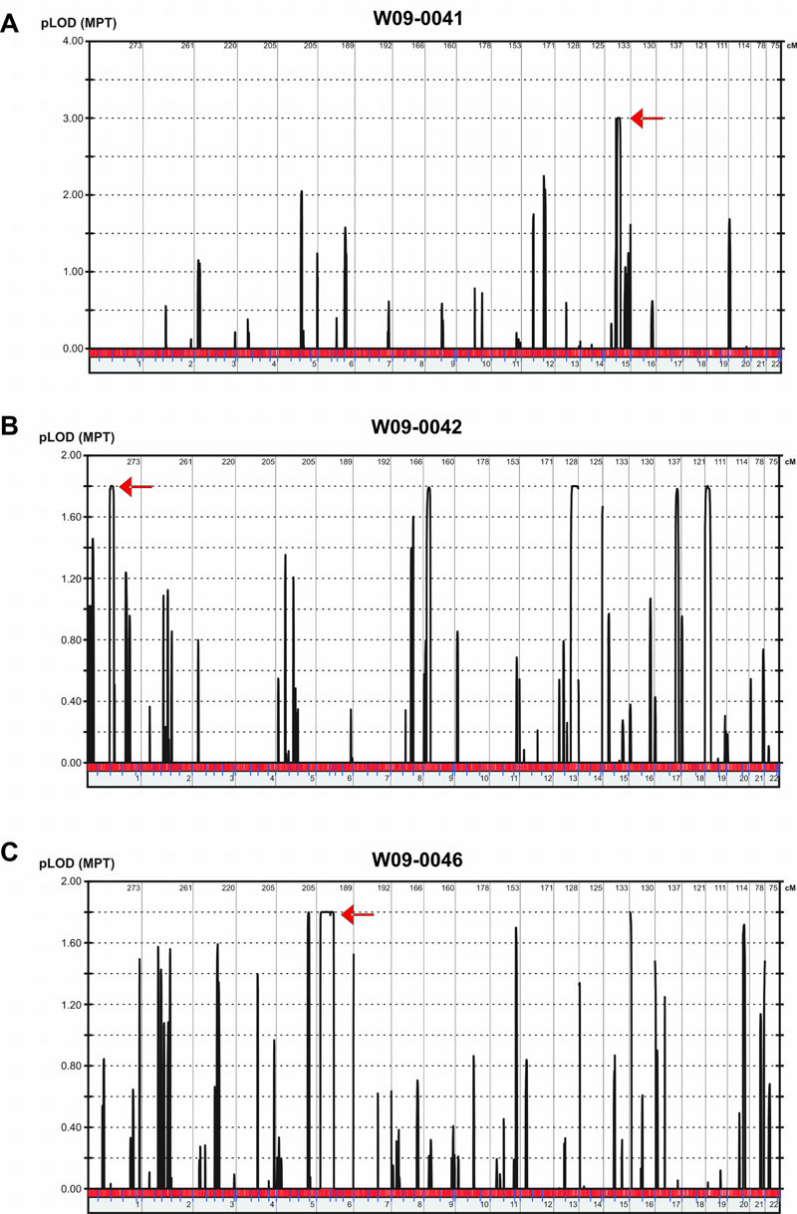
Overview of linkage plots for the three consanguineous Indonesian families. The graphs depict the linkage analysis results for three consanguineous Indonesian families, W09–0041 (**A**), W09–0042 (**B**) and W09–0046 (**C**) that were analyzed with Illumina 6k arrays. The highest LOD-score peaks were identified using EasyLinkage software. Peaks that correspond to the regions harboring the genes in which mutations were identified, are indicated with red arrows.

To determine the location of the genetic defect in the 14 presumed arRP families, affected individuals were genotyped using whole-genome SNP arrays (Figure 1). In three consanguineous families with multiple affected individuals (W09–0041, W09–0042, and W09–0046), the Illumina 6k array was used. Multipoint linkage analysis was performed to detect genomic regions that might contain the genetic defect. In family W09–0041, only a single peak with a maximum multipoint LOD score of 3.00 on chromosome 15 was identified (Figure 2A). In families W09–0042 and W09–0046, multiple peaks potentially harboring the genetic defect were identified, with multipoint LOD scores of 1.80 (Figure 2B,C). As expected in patients born from consanguineous marriages, all regions with the highest LOD scores in the three families represented continuous stretches of homozygous SNP calls (Table 2), with identical haplotypes shared between affected siblings. In the remaining 11 arRP families, high-resolution SNP genotyping was performed using Affymetrix 5.0 arrays. Homozygous regions were calculated and considered to be significant if they spanned more than 3 Mb of genomic DNA. For the families with multiple affected siblings, only those regions that were homozygous in all affected individuals were considered to potentially harbor the causative gene. In four families, no regions that were homozygous in all affected siblings were identified, whereas in a few other families, many of those regions were present. In the non-consanguineous families, the number of homozygous segments per family varied from 0 to 5, with a maximum length of 20 Mb.

**Table 2:** Homozygous regions and mutations identified in this study

**Family**	**No. of affected individuals**	**SNP array**	**Number of homozygous regions > 3Mb**	**Rank**	**Genomic position (hg18)**	**Size (Mb)**	**arRP gene in the region**	**Mutation (DNA)**	**Predicted effect (protein)**
W09–0036	3	Affy 5.0	0						
W09–0037	2	Affy 5.0	1	1	Chr 1:43.472.727–47.007.137	3.5			
W09–0038	2	Affy 5.0	**3**	**1**	**Chr 2:94.722.52–114.958.2396**	**20.2**	***MERTK***	**complex rearrangement**	**p.G654AfsX41**
				2	Chr 2: 74.821.929–86.961.722	12.1			
				3	Chr 12: 46.563.980–50.101.308	3.5			
W09–0039	1	Affy 5.0	0						
W09–0040	5	Affy 5.0	0						
W09–0041	4	Ill 6k	1	**1**	**Chr 15: 55.310.834–76.576.278**	**21.3**	***NR2E3***	**c.1025T>G**	**p.V342G**
W09–0042	2	Ill 6k	5	**1**	**Chr 1: 88.202.625–114.480.309**	**26.3**	***ABCA4***	**c.302+4A>C**	**Altered splicing**
				2	Chr 13: 97.294.596–113.158.661	15.9			
				3	Chr 9: 7.717.818–18.877.591	11.2			
				4	Chr 19: 795.02–10.879.403	10.1			
				5	Chr 17: 68.734.433–74.980.093	6.2	*PRCD*		
W09–0044	1	Affy 5.0	0						
W09–0045	4	Affy 5.0	3	1	Chr 7: 25.394.592–32.791.157	7.4			
				**2**	**Chr 2: 109.814.503–114.973.711**	**5.2**	***MERTK***	**c.2487–2A>G**	**Altered splicing**
				3	Chr 1: 49.055.999–52.858.628	3.8			
W09–0046	3	Ill 6k	3	**1**	**Chr 6: 7.426.927–76.965.256**	**69.5**	***EYS***	**c.9082G>T**	**p.D3028Y**
							*TULP1*		
				2	Chr 5: 154.427.149- 164.983.908	10.6			
				3	Chr 16: 37.354–4.720.263	4.7			
W09–0047	2	Affy 5.0	2	**1**	**Chr 5: 139.038.773–163.355.069**	**24.3**	***PDE6A***	**c.1675C>A**	**p.Y558X**
W09–0048	1	Affy 5.0	2	**1**	**Chr 1: 188.030.378–207.318.912**	**19.3**	***CRB1***	**c.3914C>T**	**p.P1305L**
			2	12	Chr 2:82.720.660–101.627.801	18.9			
W09–0049	2	Affy 5.0	5	1	Chr 9: 14.485.574–27.392.109	12.9			
				2	Chr 12: 95.081.092–104.387.559	9.3			
				3	Chr 2: 113.783.799–121.286.558	7.5			
				4	Chr 2: 60.823.051–64.299.067	3.5	*FAM161A*		
				5	Chr 1: 48.779.373–52.133.652	3.4			
W09–0050	2	Affy 5.0	21	1	Chr 1: 71.823.794–120.992.603	49.2	*ABCA4, RPE65*		
				2	Chr 1: 196.164.119–224.123.536	28	*USH2A*		
				3	Chr 5: 106.422.150–131.638.131	25.2			
				4	Chr 13: 30.509.319–52.819.567	22.3			
				5	Chr 4: 173.631.572–191.167.888	17.5			
				6	Chr 4: 121.482.239–136.825.720	15.3			
				7	Chr 1: 148.152.207–161.819.282	13.7			
				8	Chr 16: 55.072–12.523.392	12.5			
				9	Chr 17: 6.888–9.800.824	9.8	*PRCD*		
				10	Chr 12: 61.88–8.589.738	8.5			

Overview of the homozygous regions per family, and the mutations identified in this study. All genes within the regions have been screened for mutations, and all mutations that have been identified are novel. For family W09–0050, only the 10 largest homozygous regions are depicted.

### Mutation analysis of autosomal recessive RP genes in homozygous regions

In the majority of the families, one or more homozygous regions contained one of the 31 known arRP genes. Sequence analysis of these genes in the corresponding families revealed seven novel mutations likely to be causative for RP in the respective families (Table 2). The pathogenicity of the missense and splice site mutations were evaluated using a detailed in silico analysis (Table 3).

**Table 3:** Pathogenicity predictions for missense and splice site mutations.

**Amino acid change prediction**							
**Family code**	**Gene affected**	**Mutation (DNA)**	**Mutation consequence**	**SIFT**	**PolyPhen**	**Grantham score**	**PhyloP**
W09–0048	*CRB1*	c.3914C>T	p.P1305L	tolerated	probably damaging	98	4.3
W09–0046	*EYS*	c.9082G>T	p.D3028Y	not tolerated	probably damaging	160	3.6
W09–0041	*NR2E3*	c.1025T>G	p.V342G	not tolerated	probably damaging	109	0.6
**Splicing prediction**							
**Family code**	**Gene affected**	**Mutation (DNA)**	**Mutation consequence**	**GeneSplicer**	**MaxEntScan**	**NNSPLICE**	**SpliceSite finder-like**
W09–0042	*ABCA4*	c.302+4A>C	altered splicing	40%	24%	0.04%	12.3%
W09–0045	*MERTK*	c.2487–2A>G	altered splicing	100%	100%	100%	100%

List of missense and splice site mutations identified in this study and predictions of their consequences with the use of freely available software. Splicing prediction shows the percent decrease in comparison to the original splice site scores.

**Figure 3:**
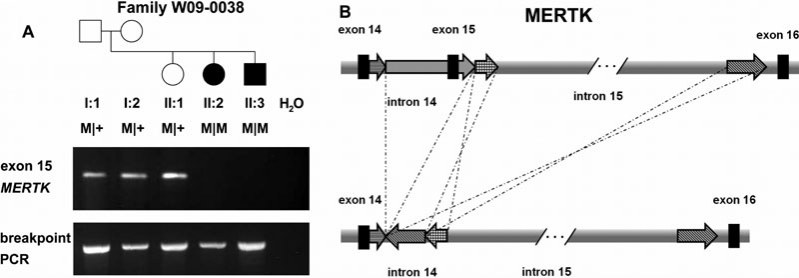
Molecular genetic analysis of MERTK in family W09–0038. **A**: In the upper panel, PCR analysis of exon 15 of *MERTK* is shown. Exon 15 was not amplified in the two affected individuals of family W09–0038. All relatives and their position in the pedigree are indicated above the electropherogram. Lower panel: after identification of the breakpoints of the complex rearrangement, PCR primers were designed to amplify a product spanning the deletion. A PCR product indicating the presence of a rearrangement is observed in all individuals demonstrating that the unaffected family members are heterozygous carriers of the deletion. **B**: Schematic representation of the complex rearrangement in MERTK. A deletion of a genomic region containing exon 15 is accompanied by a duplication and an inversion event.

In family W09–0038, the largest homozygous region contained the c-mer proto-oncogene tyrosine kinase (*MERTK*) gene. Exon 15 failed to amplify in the affected individuals but not in the unaffected family members, suggesting a genomic deletion (Figure 3A). Detailed analysis revealed the occurrence of a complex rearrangement that included a 1732-bp deletion containing exon 15 of *MERTK* (Figure 3B). The absence of exon 15 from *MERTK* mRNA is predicted to result in a frameshift and premature stop codon, which may lead to truncation of the MERTK protein (p.G654AfsX41) or trigger nonsense-mediated mRNA decay. The affected individuals of family W09–0045 displayed three shared homozygous regions, of which the second largest contained *MERTK*. Sequence analysis revealed a homozygous mutation (c.2487–2A>G) that affects the consensus splice site of exon 19 and most likely results in aberrant splicing and premature termination of the MERTK protein, including the highly conserved tyrosine kinase domain (Table 3).

**Figure 4:**
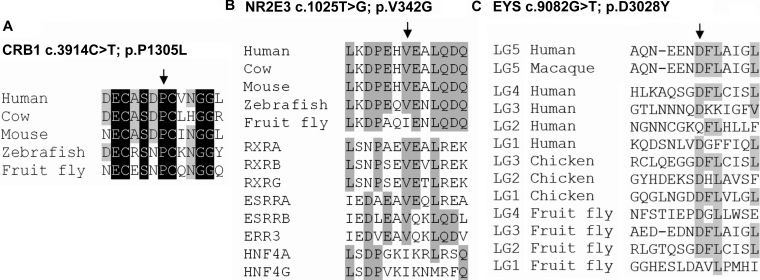
Sequence comparison of amino acids mutated in Indonesian RP families. The mutated and flanking amino acids, from orthologous and homologous protein sequences, for **A**: CRB1 **B**: NR2E3 and **C**: EYS. The arrows indicate the position of the mutated amino acid residue in the alignment. Residues that are conserved in all protein sequences are depicted in white on a black background, whereas residues that are conserved in more than 50% of the analyzed sequences are indicated in black on a gray background.

In family W09–0041, four affected siblings shared only one large homozygous region, on chromosome 15, harboring the nuclear receptor subfamily 2, group E, member 3 (*NR2E3*) gene. Mutation analysis of *NR2E3* identified a homozygous mutation substituting a glycine for a valine residue (c.1025T>G; p.V342G). The PolyPhen-2 program predicts this variant to be probably damaging, and SIFT indicates that in this position only a valine or isoleucine is tolerated (Table 3). Furthermore, the mutation was homozygously present in five affected family members and was absent or heterozygously present in three unaffected siblings. Finally, this variant was not detected in 298 ethnically matched control alleles. *NR2E3* encodes a transcription factor that belongs to the family of nuclear hormone receptors, with a DNA-binding domain (DBD) at the N-terminus and a ligand-binding domain (LBD) located more C-terminally [15]. Most *NR2E3* mutations that cause retinal dystrophy affect amino acid residues located in these two domains [16]. The LBD of NR2E3 is structurally similar to that of other nuclear hormone receptor family members and consists of 12 structurally conserved α-helices [17,18]. The valine residue at position 342 is highly conserved, both in the NR2E3 proteins of other species and in the LBD of other nuclear hormone receptors, being either a valine or a similar isoleucine residue in all other protein sequences (Figure 4B). This high degree of conservation suggests that a relatively large nonpolar amino acid must be present at this position to preserve the structure and/or function of the LBD of these proteins. The glycine residue that is present in the mutant NR2E3 protein is small and might interrupt the structure and render the protein inactive or partially active. The pathogenic character of this variant is supported by the fact that this alteration has not been found in alleles of healthy ethnically matched individuals.

In family W09–0042, the largest homozygous region contains the ATP-binding cassette, sub-family A, member 4 (*ABCA4*) gene, which has been associated with Stargardt's disease [19], cone-rod dystrophy, and to a lesser extent, RP [20]. Sequence analysis revealed a nucleotide variant in the splice donor site of exon 3 (c.302+4A>C) homozygously present in the two affected siblings and absent or heterozygously present in their nonaffected relatives. In addition, the variant was not detected in 298 ethnically matched control chromosomes. In silico prediction of the strength of the splice donor site showed a decrease due to the alteration (e.g., 40% decrease in GeneSplicer, 24% in MaxEntScan, 12.3% SpliceSiteFinder-like, and 0.04% decrease in NNSPLICE), indicating that this variant might alter *ABCA4* splicing (Table 3). However, due to the unavailability of patient RNA or lymphoblastoid cell lines, altered *ABCA4* splicing could not be confirmed in vivo. Hence, the pathogenicity of this variant remains uncertain.

In family W09–0046, three regions that are homozygously shared by two affected siblings were detected. The second largest region contained two known arRP genes, namely tubby-like protein 1 (*TULP1*) and eyes shut homolog (Drosophila) (*EYS*). Sequence analysis of *TULP1* did not reveal any pathogenic variants, whereas a missense variant was detected in *EYS* that segregated with arRP in the family (c.9082G>T; p.D3028Y). This alteration affects an aspartic acid residue localized in the fifth laminin A G-like domain of the human EYS protein. The aspartic acid residue at this position is also present in homologous positions of other EYS LG domains and is evolutionarily conserved in EYS proteins of other species (Figure 4C). Both SIFT and PolyPhen-2 predict this change to be damaging, which is further supported by high Grantham and PhyloP scores (Table 3). Intriguingly, a similar mutation (p.D2767Y), located at the homologous position in the fourth laminin A G-like domain of EYS, was recently found in a Pakistani family segregating arRP [21]. This suggests that the aspartic acid residue located at that position within the laminin A G-like domain is crucial for proper functioning of EYS and that the p.D3028Y mutation is indeed pathogenic in family W09–0046.

In two affected siblings from family W09–0047, only a single homozygous region was detected that harbored the phosphodiesterase 6A, cGMP-specific, rod, alpha (*PDE6A*) gene. A nonsense mutation (c.1675C>A; p.Y558X) was found to segregate with RP in this family, and this is predicted to either result in premature termination of the α subunit of the phosphodiesterase enzyme or to cause nonsense-mediated decay of *PDE6A* mRNA.

The largest homozygous region detected in the affected individual 50068 from family W09–0048 contained the crumbs homolog 1 (*CRB1*) gene, and sequence analysis revealed a transition of cytosine to thymine, resulting in the substitution of a leucine for a proline residue (c.3914C>T; p.P1305L). This alteration was not detected in 149 ethnically matched control individuals and is predicted to be probably damaging by PolyPhen, although the SIFT software predicts it to be tolerated (Table 3). The proline residue that is substituted resides in one of the EGF-like calcium-binding domains of CRB1 and neighbors one of the six core cysteine residues that are essential for the formation of disulphide bridges and proper structure of these domains [22]. The fact that the proline residue is highly conserved throughout vertebrate evolution and even in fruit fly (Figure 4A), suggests that this mutation is causative for arRP in this family.

In two of the families (W09–0049 and W09–0050), genes residing in the largest homozygous regions (family with sequence similarity 161, member A [*FAM161A*], *ABCA4,* retinal pigment epithelium-specific protein 65 kDa [*RPE65*], Usher syndrome 2A (autosomal recessive, mild) [*USH2A*], and progressive rod-cone degeneration [*PRCD*]) were excluded. Four families (W09–0036, W09–0039, W09–0040, and W09–0044) had no significant homozygous regions.

For the families with plausible X-linked inheritance (W09–0048, W09–0049 and W09–0050), we performed screening of the two known X-linked RP genes. *RP2* and *RPGR* were sequenced, but no mutations were identified.

## Discussion

In this study we analyzed the molecular genetic causes of RP in 14 Indonesian families with an apparent recessive mode of inheritance; especially in the simplex families, de novo mutations or an X-linked inheritance cannot be ruled out.

In all 14 families, genome-wide homozygosity mapping was performed to find genomic regions potentially harboring the causative genetic defect. In ten of the 14 families, significant homozygous regions were detected in the affected individuals, ranging from 3.4 to 69.5 Mb in size (Table 2). On average, European populations, which are mostly outbred, display a total of about 93 Mb of genomic runs of homozygosity (ROH) longer than 0.5 Mb, corresponding to approximately 3.5% of the genome [23]. Unfortunately, no such studies were conducted within the Indonesian population. In our small cohort, these average ROH in 16 individuals from nine families without reported consanguinity (see Figure 1) comprised 146 Mb (~5.5%) of genomic DNA, which suggests a slightly higher level of inbreeding in this population. Consanguineous unions within the Indonesian culture are not uncommon. Moreover, Indonesia consists of a large archipelago of islands, 922 of which are inhabited, which further increases the possibility of marrying distant relatives. Hence, our data support the hypothesis that the Indonesian population is highly suited for a homozygosity mapping approach to determine genetic defects underlying recessive diseases.

In nine families, the homozygous regions contained one or more of the 31 known arRP genes. Sequence analysis of all these genes in the corresponding probands revealed potentially pathogenic mutations in seven families. A homozygous nonsense mutation in *PDE6A*, a canonical splice mutation in *MERTK*, and a complex rearrangement in *MERTK* are three mutations that are undoubtedly pathogenic in the respective families. The causality of the novel three missense and one splice donor mutation is not obvious. The pathogenic effect of missense alterations may be difficult to interpret in the absence of suitable functional assays or animal models. However, due to the high degree of conservation of the mutated amino acid residues, their location within predicted functional domains of the encoded proteins, and the absence of these alleles in ethnically matched control individuals, the missense variants in *CRB1*, *EYS,* and *NR2E3* are considered to be the probable pathogenic mutation in the respective families. The splice alteration in *ABCA4* (c.302+4A>C) was also not detected in Indonesian controls, and the strength of the splice donor site was predicted to be decreased by several splice-site strength prediction programs. The fourth nucleotide of an intron in primates is an adenine in 71% of the cases, a guanine in 12%, and a cytosine in only 8% [24]. Therefore, the strength of the splice donor site in *ABCA4* may be reduced due to the A-to-C transition at that position. However, reverse transcriptase (RT)-PCR analysis on patient-derived RNA is necessary to confirm pathogenicity of this variant in the respective family.

The identification of genetic events underlying RP was previously clinically useful only in genetic counseling. However, with the successful development of gene therapy for retinal dystrophies, such as for Leber congenital amaurosis due to *RPE65* mutations [25-27], molecular diagnosis is of increasing importance to enroll patients in future clinical trials. Since RP is a progressive disease, early diagnosis is essential, and the identification of a causative alteration will be crucial in the near future to plan gene- or even mutation-tailored therapy. Homozygosity mapping has been demonstrated to be a powerful tool to detect recessive mutations underlying RP in the Indonesian population. Based upon the criteria of the percentage of homozygous regions in an individual genome, which is about 50% higher than in outbred European populations, SNP array analysis appears to be the most practical approach. Since we have identified the probable causative genetic defect in seven out of 14 arRP families, using homozygosity mapping, and excluded *RPGR* and *RP2* in families suggestive of X-linked inheritance, some of the other families might harbor a homozygous mutation in a novel arRP gene, in any of their homozygous regions. However, many of the genetically unsolved families are non-consanguineous and do not display large homozygous regions. Therefore, the majority of these families are likely to carry compound heterozygous mutations, either in one of the known arRP genes or in yet to be identified genes. Only next-generation sequencing approaches, either on predefined genomic regions [28] or unbiased exome sequencing [29,30], will allow a cost- and time-efficient analysis to discover the genetic causes underlying RP in the remaining families.

Due to the extreme heterogeneity of arRP, it is not easy to predict the percentage of arRP cases that can be explained by mutations in the known arRP genes, although current estimates are around 50%. Being aware of the relatively small size of our cohort but taking into consideration that some of the 14 tested arRP families may carry compound heterozygous mutations in known arRP genes or homozygous mutations in these genes in homozygous regions smaller than 3 Mb, we can conclude that the known arRP genes appear to explain at least 50% of the underlying genetic defects. This conclusion will be strengthened upon the analysis of more families, both in the Indonesian and in other populations.

Taken together, the results described in this study have helped to clarify the molecular genetic causes underlying recessive RP in Indonesia and as such will be beneficial in terms of genetic counseling, disease diagnosis, and/or disease prognosis and in the future might help to select patients eligible for genetic therapies to cease the progression of this disabling disease.
